# Metals – impact and implications

**DOI:** 10.2478/v10102-010-0039-6

**Published:** 2010-11

**Authors:** Max Vojtíšek, Jana Patková, Jana Knotková, Lucie Kašparová, Miroslava Hornychová, Emil Frantík, Jaroslav Formánek, Eva Švandová

**Affiliations:** National Institute of Public Health, Šrobárova 48, Prague 10, 10042, CZECH REPUBLIC

**Keywords:** metals, oxygen consumption, blood-brain barrier

## Abstract

Impact of metal *in vitro* administration on rat tissue oxygen consumption is referred in the first part. Toxicological implications of *in vivo* metal administration to rats and the study of potential penetration of metal into the rat brain, which may eventually result in oxygen radical production are presented in second part.

## Introduction

It is well known fact, that a series of metals are included within enzymatic reactions as important cofactors but many others have no obvious biological role and at certain levels may manifest noxious effects.

The aim of this communication was to depict few aspects of metal toxicology. Previous in vitro studies have shown, that elementary process, like oxygen consumption can be diferrentially influenced by cadmium, a metal which is not presumed to belong to essential elements (Vojtíšek, 1983). Following second part presents a comparison of penetration potential of several metals: cadmium, lead and manganese, through the blood-brain barrier.

Implications, concerning physiological mechanisms of metals within tissues are postulated, especially those, relevant to cellular oxidative metabolism. In vitro cadmium is known to increase O_2_^–^ production in human granulocytes or alveolar macrophages (Amoruso and Witz, 1981). Desoize (2003) is including chromium, arsenic, nickel, vanadium, iron, copper and manganese as metals with action, realised by ROS production. Lead is referrred in connection with oxidative stress by Soltaninejad *et al*. (2003).

Penetration of some of above mentioned metals to the brain (as well as other organs) may implicate similar toxicologic impact. Penetration of manganese or cadmium to the brain was repeatedly evaluated (Vojtíšek *et al*., 1989, 2005). Tests with lead supplement a triad of tested metals.

## Methods

### Oxygen consumption

Wistar female rats, kept on standard diet, water ad lib. were used. Oxygen consumption was measured with Clark electrode on mucosal preparations of small intestine. Cadmium was added in vitro to glass polarographic chamber in concentration range from 10^–2^ to 10^–6^ mol/l (Vojtíšek, 1983).

### Analysis of metal brain retention after *in vivo* exposure

Wistar female rats, kept on standard diet, water ad lib. were used. In case of cadmium (Cd) rats were intratracheally (IT) administered a single dose of 43.28 µg Cd/ kg b.wt., in case of lead IT dose of 1.36 mg Pb/kg b.wt., and in case of manganese (Mn) rats were given IT dose of 2.88 mg Mn/kg b.wt., respectively. Within subchronic tests with manganese a dose was given twice a week for 12 weeks.

Samples of the mineralized brains of rats were analyzed by means of atomic absorption spectrometer AA30 Varian with graphite tube GTA 96, Zeeman background correction and autosampler for GTA 96 (XX). There were metal exposed and metal non-exposed control groups of rats for the spectrometric analysis of individual metal. There were usually 8–10 samples from both groups of rats, metal exposed or control animals.

Institutional as well as national guides for the care and use of laboratory animals were strictly followed.

## Results

### Oxygen consumption

Inhibition of mucosal oxygen consumption of ileum was proportional to the molar concentration of cadmium used *in vitro*. High level of cadmium concentration of 2.2×10^–2^ mol/l caused 62.4±4.9% decrease of respiration, while 4.5×10^–3^ cadmium level resulted in 31.9±8.1% decrease only (p<0.01 and p<0.05, respectively, for n=6, Student′s t-test). With the administration of lower cadmium levels from 10^–4^ up to 10^–6^ mol/l there were no significant respiration stimulations observed.

### Analysis of metal brain retention after *in vivo* exposure

Analysis of brain metal retentions is depicting penetration potential of individual metals tested: Cd, Pb and Mn to cross the blood-brain barrier. Individual metal concentration levels, which succeeded to enter the central nervous system area are following: 0.81±0.13 ng Cd/g tissue as related to <0.5ng Cd/g tissue for unexposed controls and 0.016±0.005 µg Pb/g tissue as related to <0.01 µg Pb/g tissue for unexposed controls. The last metal tested was manganese. Metal analysis have shown 0.59±0.04 µg Mn/g tissue as related to 0.38±0.03 µg Mn/g tissue for unexposed controls. The results for manganese brain retention are statistically significant (p<0.05) while in case of cadmium and lead spectrometric analysis was at the lowest detection limit, which is obvious from retention of control unexposed groups of rats. Subchronic longterm administration of Mn succceeded to increase the brain manganese level up to 0.92±0.05 µg Mn/g tissue as related to 0.51±0.02 µg Mn/g tissue for unexposed controls.

## Discussion

This work presents two aspects of metal interference with homeostatic conditions in mammal tissues. On one hand metal can interfere with processes of oxidative metabolism, specifically with oxygen consumption of various tissues (Vojtíšek *et al*., 1983, 1989). Contemporary data comment possible mechanism of Cd ions effects within tissues and subcellular structures, e.g. Cd^2+^ stimulation of mitochondrial respiration, enhanced in presence of thiol groups (Belayeva *et al*., 2004) or cadmium direct influence on the dysfunction of isolated mouse liver mitochondria, including the inhibition of respiration and release of cytochrome c (Li *et al*., 2003).

Another aspect of metal tissue presence is potential connection with the over-production of reactive oxygen radicals, especially within central nervous system. Updated information on iron, copper, cobalt, vanadium, cadmium, arsenic and nickel reports on their capacity of free radical formation (Valko *et al*., 2006). Attention was paid therefore to observe the penetration of several metals with neurotoxic potential: Cd, Pb and Mn into the rat brain.

Intentionally a single non-toxic dose, administered intratracheally was selected in the study of penetration of 3 metals observed to model inhalatory port of entry. Because of relatively thin alveolar barrier before the penetration of noxa to blood system this administration represents an access comparable to intravenous administration. Differentiated defence capacity of the blood-brain barrier against the entry of potentially toxic metals was observed. There is evidence indicating rather difficult penetration of some tested metals, like cadmium, across the blood-brain barrier (Vojtíšek *et al*., 2007).

It is interesting to observe the control level of brain manganese of unexposed rats in subchronic test, which is aproaching the level of Mn treated rats within acute test. It can be explained because of essential role of manganese in the majority of mammal tissue, where its is included in superoxide dismutase and other enzymatic systems. This level – biological background is gradually increasing with age of rats in subchronic longterm experiment.

Preliminary experiments strive to discern some implications of oxidative stress within the brain as a result of metal – Pb penetration across the blood-brain barrier and disruption of homeostatic mechanisms, preventing such situation.
